# 5-Aminolevulinic Acid False-Positive Rates in Newly Diagnosed and Recurrent Glioblastoma: Do Pseudoprogression and Radionecrosis Play a Role? A Meta-Analysis

**DOI:** 10.3389/fonc.2022.848036

**Published:** 2022-02-17

**Authors:** Luca Ricciardi, Carmelo Lucio Sturiale, Alba Scerrati, Vito Stifano, Teresa Somma, Tamara Ius, Sokol Trungu, Michele Acqui, Antonino Raco, Massimo Miscusi, Giuseppe Maria Della Pepa

**Affiliations:** ^1^ Division of Neurosurgery, Sant’Andrea Hospital, Department of Neuroscience, Mental Health and Sense Organs (NESMOS), Sapienza University of Rome, Rome, Italy; ^2^ Institute of Neurosurgery, Fondazione Policlinico Universitario A. Gemelli, Rome, Italy; ^3^ Division of Neurosurgery, Catholic University of Rome, Rome, Italy; ^4^ Neurosurgery Department, S. Anna University Hospital, Ferrara, Italy; ^5^ Department of Morphology, Surgery and Experimental Medicine, University of Ferrara, Ferrara, Italy; ^6^ Division of Neurosurgery, Department of Neurosciences and Reproductive and Odontostomatological Sciences, Federico II University, Naples, Italy; ^7^ Division of Neurosurgery, Neuroscience Department, University Hospital of Udine, Udine, Italy; ^8^ Neurosurgery Unit, Cardinal G. Panico Hospital, Tricase, Italy

**Keywords:** glioblastoma, high-grade glioma (HGG), recurrent glioblastoma, 5-ALA fluorescence, pseudoprogression, radionecrosis

## Abstract

**Background:**

Several studies have confirmed the impact of 5-aminolevulinic acid (5-ALA) on the extent of resection in newly diagnosed glioblastoma (GBM). However, there are controversies on the 5-ALA fluorescence status in recurrent GBM surgery, with specific reference to pseudoprogression or radionecrosis; therefore, the safety and accuracy of surgical planning in 5-ALA-assisted procedures in the recurrent context are still unclear.

**Materials and Methods:**

This is a systematic review and meta-analysis of comparative studies on the use of 5-ALA in newly diagnosed and recurrent GBM, consistently conducted according to the Preferred Reporting Items for Systematic Reviews and Meta-Analyses (PRISMA) statement. Data on fluorescence status and correlation between fluorescence and histological findings were collected. We performed a meta-analysis of proportions to estimate the pooled rates of each outcome.

**Results:**

Three online medical databases (PubMed, Scopus, Cochrane Library) were screened, 448 articles were evaluated, and 3 papers were finally included for data analysis. Fluorescence rate was not different between newly diagnosed and recurrent GBM [p = 0.45; odds ratio (OR): 1.23; 95% CI: 0.72–2.09; I^2^ = 0%], while the rate of 5-ALA fluorescence-positive areas not associated with histological findings of GBM cells was higher in recurrent GBM (p = 0.04; OR: 0.24; 95% CI: 0.06–0.91; I^2^ = 19%). Furthermore, there were no cases of radionecrosis in false-positive samples, while inflammation and signs of pseudoprogression were found in 81.4% of the cases.

**Discussion and Conclusions:**

Therefore, a robust awareness of 5-ALA potentialities and pitfalls in recurrent GBM surgery should be considered for a cognizant surgical strategy. Further clinical trials could confirm the results of the present meta-analysis.

## Introduction

An extended microsurgical resection over the anatomical limits of the solid lesion on contrast-enhanced T1-weighted MRI images is currently established as a paramount determinant in terms of both overall survival (OS) and progression-free survival (PFS) similarly in newly diagnosed and recurrent glioblastoma (GBM) (1, 2). Since the approval of 5-aminolevulinic acid (5-ALA) for medical use in 2007 in Europe and in 2017 in the United States, several studies have confirmed its impact on the extent of resection rate in GBM surgeries. Therefore, 5-ALA has been progressively adopted as a standard tool in neurosurgical procedures for GBM, providing valuable clinicoradiological outcomes (1, 3–5).

After its administration, 5-ALA mainly accumulates in malignant glial cells, where it is converted to fluorescent protoporphyrin IX (PpIX). However, it should be carefully considered that PpIX can also be found in non-tumoral structures, such as ependymal cells (6, 7). Furthermore, the presence of inflammatory tissue, such as in the case of peritumoral reactive inflammation, pseudoprogression (PP), or radiation-induced necrosis [radionecrosis (RN)] may influence the intraoperative fluorescence detection (8, 9). Therefore, surgeons must be aware that *not everything that glitters is gold*. Hence, a critical analysis of the clinicoradiological outcomes of 5-ALA-guided surgery in recurrent gliomas may help shed light on this (8).

The aim of the present systematic review and meta-analysis of comparative studies, reporting the 5-ALA fluorescence status in newly diagnosed and recurrent GBM, is to investigate true-positive and false-positive fluorescence rates, histological findings in 5-ALA-positive samples with no evidence of GBM cells, and the fluorescence status of RN and PP areas.

## Materials and Methods

### Study Design

The present investigation is a systematic review and meta-analysis conducted according to the Preferred Reporting Items for Systematic Reviews and Meta-Analyses (PRISMA) statement.

### Search Strategy

The review question was formulated according to the PICO (P: patients; I: intervention; C: comparison; O: outcomes) scheme, as follows: in case of newly diagnosed or recurrent glioblastoma multiforme (P), is 5-ALA (I) a useful tool for increasing the extent of resection (O), considering the tumor fluorescence positivity and the false-positive rates (C)?

Three different medical databases (PubMed, Scopus, and Cochrane Library) were screened using the following search terms: “5-ALA”, “aminolevulinic”, “recurrent glioma”, “recurrent glioblastoma”, “radionecrosis”, “pseudoprogression”, “glioma recurrence”, “glioblastoma recurrence”, “radiation necrosis” [MeSH], combined using Boolean operators “AND” and “OR”.

Titles and abstracts were screened in the first search round. In the second round, full text of eligible papers and their reference lists (forward search) were evaluated. Then, papers were considered for data reporting and availability in the third round of search. Studies matching our inclusion criteria were finally included in the present systematic review and meta-analysis. Two authors (GDP and AS) independently conducted the first two search rounds, and any discordance was solved by consensus with a third senior author (LR).

### Inclusion and Exclusion Criteria

Comparative studies in English language on newly diagnosed and recurrent GBM, reporting data on the intraoperative 5-ALA fluorescence status (positive/negative), and histological findings of 5-ALA-positive regions were considered for eligibility. True positive was considered as 5-ALA-positive fluorescence and histological confirmation of GBM; false positive, instead, as 5-ALA-positive fluorescence and no histological diagnosis of GBM. Reviews, case reports, letters, technical notes, video articles, and studies on pediatric population (<18 years old) were not considered. The inclusion and exclusion critera are summarized in **Table 1**.

**Table 1:** Inclusion and exclusion criteria.

Inclusion criteria	Exclusion criteria
• English language • Comparative studies on 5-ALA fluorescence rate in newly diagnosed and recurrent glioblastoma • Histological findings in case of false positivity • Adult population	• Reviews, clinical case, editorial, technical notes studies • Pediatric population (<18 years) • Published prior to 2007

### Data Extraction

Included studies were screened for the number of newly diagnosed and recurrent GBM patients, their demographics, type of operative microscope, and percentage of true and false positives in each group. Furthermore, histological findings in case of false-positive samples were also collected.

### Statistical Analysis

We performed a meta-analysis of proportions to estimate the pooled rates of each outcome. Proportion meta-analyses were not used when the frequency of an outcome was reported in <1% of the sample [raw proportions and 95% confidence interval (95% CI) were reported in such cases], and a random-effects model was adopted to account for the inter-study heterogeneity.

## Results

### Study Selection

**Figure 1:**
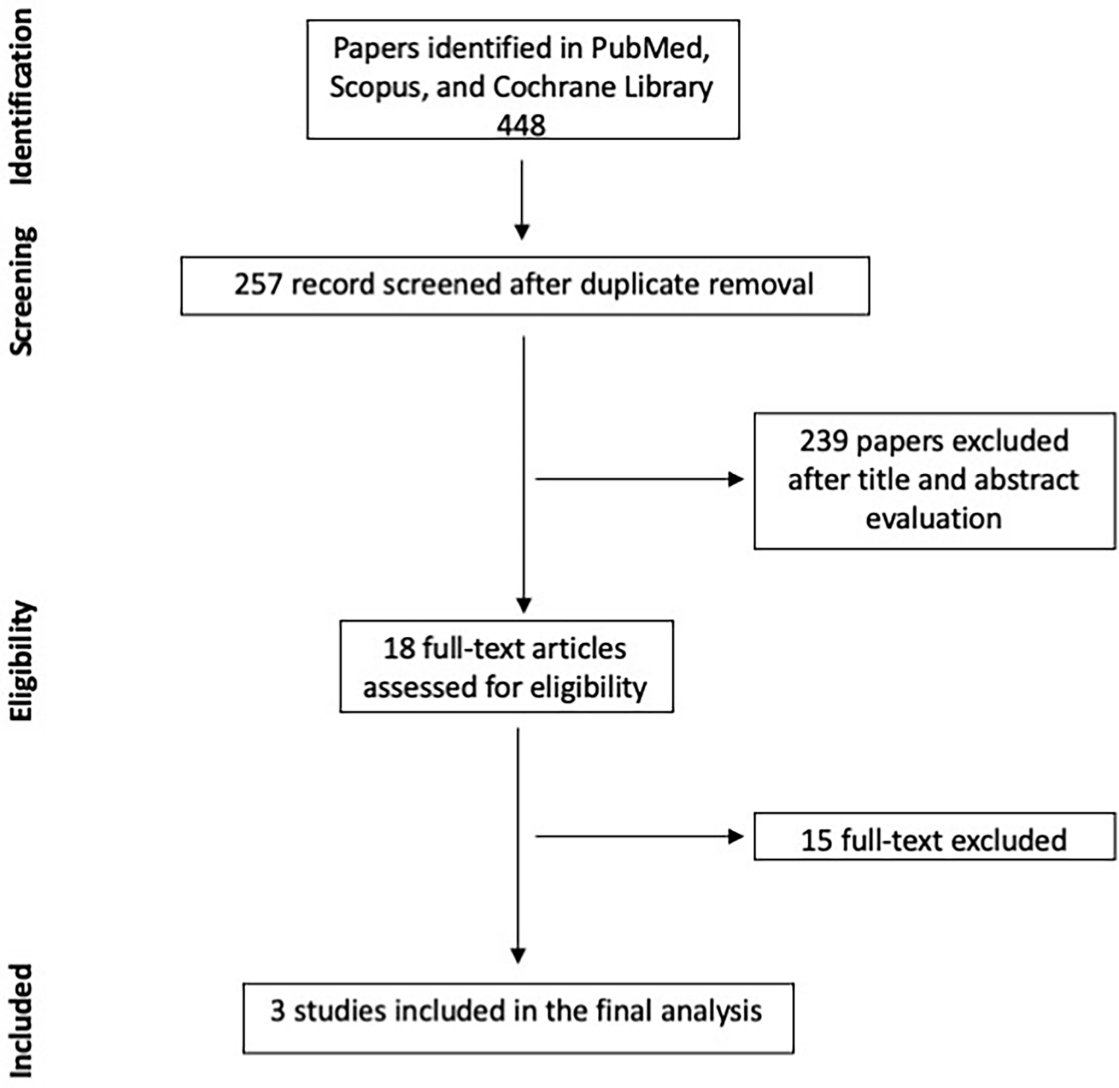
Search strategy.

A total of 448 titles and abstracts were firstly screened. Eighteen papers were considered as eligible, and their full text was evaluated. After the full-text analysis and the forward search, 3 papers were finally included for meta-analysis. The search strategy is summarized in **Figure 1**. Papers excluded with reason after their full-text examination are summarized in **Supplementary Table S1**.

### Included Studies

We included 331 patients from 3 studies, 1 retrospective (10) and 2 prospective (11, 12).

A newly diagnosed GBM was reported in 212 patients while a recurrent GBM in 119. Patient’s gender and age were reported in 1 out of the 3 (33%) included studies.

All patients received histological diagnosis according to the WHO 2016 guidelines. All patients who underwent second surgeries for GBM recurrence received the Stupp protocol between the two procedures.

The use of 5-ALA fluorescence was used to perform multiple biopsies in each included study, and the definition of *“false positive”* was homogeneously considered as the absence of GBM cells at least in one of the analyzed samples.

### Positive 5-Aminolevulinic Acid Fluorescence Rate

The type of operative microscope used for conducting the surgical procedures was reported in 1 out of the 3 included studies, and it was Pentero (Carl Zeiss, Oberkochen, Germany).

A positive 5-ALA fluorescence was reported in 169 (79.7%) out of the 212 newly diagnosed GBM and in 89 (74.8%) out of the 119 recurrent GBM.

**Figure 2:**
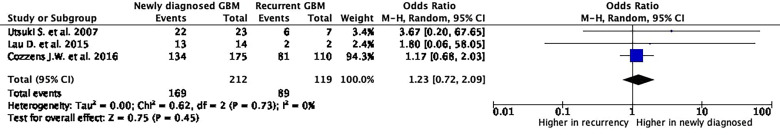
5-Aminolevulinic acid (5-ALA) fluorescence rate in newly diagnosed and recurrent glioblastoma. The Foster Plot, from the meta-analyses of proportions to estimate the pooled rates of 5-ALA-positive fluorescence rate in newly diagnosed and recurrent glioblastoma. According to the Plot, there are no significative differences comparing the pooled rates from the two meta-analytic groups (p = 0.45).

The rate of positive 5-ALA fluorescence was not significantly different between newly diagnosed and recurrent GBM [p = 0.45; odds ratio (OR): 1.23; 95% CI: 0.72–2.09; I^2^ = 0%]. The heterogeneity was very low, confirming the reliability of data (**Figure 2**).

### False-Positive 5-Aminolevulinic Acid Fluorescence Rate and Histological Findings

The histopathology did not confirm the presence of GBM cells in 7 (4.1%) out of the 169 5-ALA fluorescence-positive newly diagnosed cases and in 9 (10.1%) out of the 89 5-ALA fluorescence-positive recurrent GBM.

The incidence of false positives was significantly higher in recurrent GBM cases (p = 0.04; OR: 0.24; 95% CI: 0.06–0.91; I^2^ = 19%) with a relatively low heterogeneity rate.

**Figure 3:**
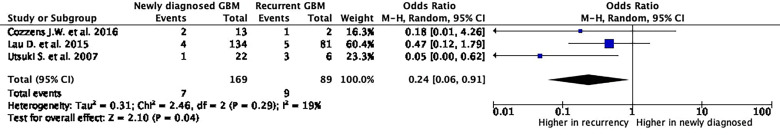
5-Aminolevulinic acid (5-ALA) fluorescence-positive areas non-associated with histological finding of glioblastoma cells. The Foster Plot, from the meta-analyses of proportions to estimate the pooled rates of 5-ALA-positive fluorescence areas non-associated with histological finding of glioblastoma cells, defined in the paper as false-positive areas. According to the Plot, a significantly higher false-positive rate occurs in recurrent glioblastoma.

Histological report was available in 13 (81.3%) out of the 16 false-positive cases. It reported “abnormal brain tissue characterized by reactive astrocytes and scattered inflammatory cells” in 11 (68.8%), “normal brain tissue” in 1 (6.3%), “infiltrating neutrophils” in 1 (6.3%), and not reported in 3 (18.8%). No cases of RN were reported in false-positive cases among the recurrent GBMs (**Figure 3**).

## Discussion

The present systematic review and meta-analysis demonstrates no significant differences in terms of positive 5-ALA fluorescence rates between newly diagnosed and recurrent GBM, while the rate of 5-ALA-positive areas not associated with histological finding of GBM cells is significantly higher in recurrent GBM surgeries (**Figures 2**, **3**).

Our data analysis also confirmed that 5-ALA accumulates in malignant glial cells in both newly diagnosed and recurrent GBM, showing that there are no differences between newly diagnosed and recurrent GBM in terms of intraoperative 5-ALA-positive fluorescence rates. This implies that 5-ALA can be considered a useful and reliable tool for identifying GBM cells during microsurgical procedures. Furthermore, the accumulation of 5-ALA in peritumoral inflammatory areas also leads surgeons to conduct a supramarginal resection, which is a pursuable outcome in GBM surgeries.

Conversely, in literature and in general practice, its indisputable role has been well underlined for newly diagnosed high-grade glioma (HGG) cases (13, 14). On the other hand, its significance and possible pitfalls in recurrent HGGs are less delineated; potentialities and drawbacks in the recurrent setting are instead less clear (15).

Recent literature, indeed, focused on false-negative conditions specifically addressing the issues of PP and RN in HGGs (16, 17). Recurrent GBMs represent different entities from newly diagnosed ones in terms of peritumoral peculiar findings, such as RN and PP. Therefore, 5-ALA fluorescence status in recurrent GBMs and their peritumoral area may be influenced by the aforementioned modifying factors. This should be carefully considered when planning 5-ALA-assisted procedures in recurrent GBM to avoid resections improperly exceeding planned limits.

Our results showed that the rate of 5-ALA-positive areas with no GBM cells in their context, classified as false-positive regions, is significantly higher in recurrent GBMs than in newly diagnosed ones (p = 0.04). A critical analysis of this finding could aim to better understand mechanisms underlying the higher false-positive rate in recurrent glioma.

### Pseudoprogression

PP is defined as peritumoral inflammatory tissue that may be erroneously reported as tumor progression or recurrence on MRI images (18). The PP phenomenon is well documented, and its MRI aspect is justified by its histological findings, consisting of inflammation, neutrophil and macrophage infiltration, reactive astrocytes, high mitotic rate, and neoangiogenesis (19). This may justify the 5-ALA fluorescence in these peritumoral areas, as confirmed in our results. In fact, *“abnormal brain tissue characterized by reactive astrocytes and scattered inflammatory cells”* was found in 68.8% of false-positive specimens and *“infiltrating neutrophils”* in 6.3%. The histological report was *“normal brain”* in 6.3%, while it was not available in 18.8% of the included cases. Accordingly, inflammatory findings were found in 82.1% of false-positive samples, confirming that PP could represent the underlying mechanism of 5-ALA accumulation in non-malignant GBM peritumoral areas.

Post-surgical or post-radiation infiltration of reactive astrocytes (responsive astrocytosis), immune cell presence, and 5-ALA extracellular accumulation can be responsible for false-positive cases. Indeed, histiocytes/macrophages (which have function of phagocytosis) and lymphocytes can internalize 5-ALA: this may lead to a significant buildup of porphyrin precursors, making the tissue fluorescent (8). Regarding 5-ALA leakage and extracellular fluorescence accumulation, the latter is true mostly for lesions with pronounced perifocal edema.

### Radiation Necrosis

RN is a relatively frequent finding in postoperative follow-up imaging from GBM patients. It consists of local necrosis and fibrous tissue spreading, usually contiguously to the surgical cavity. Although RN consists of a relatively low-activity metabolic tissue, its enlargement may determine some grade of compression on the surrounding brain tissue, resulting in inflammatory phenomena (20). Conversely, RN is not supposed to be 5-ALA positive due to its metabolic status and histological characteristics (8).

This meta-analysis confirms this evidence, as no cases of RN were reported among false-positive samples.

These data are of importance, as there is not a clear picture about its behavior in literature with 5-ALA. Indeed, an aspect to be critically appraised concerning this issue, especially in oldest reports, is the fact that neuropathological examination in many reports could underestimate the presence of tumor cells. As a matter of fact, in many cases, hematoxylin–eosin staining depicting reactive changes only can be actually converted to the diagnosis of infiltrating tissue after additional immunohistochemistry investigations. Furthermore, while RN can occur in a relatively small area, the surrounding tissue has been radiated itself, then some grade of radiation-induced inflammation has to be considered. Therefore, while proper RN tissue should not display fluorescence, the surrounding brain may be characterized by inflammatory phenomena and histologically related findings and hence might display some degree of 5-ALA fluorescence.

### Surgical Considerations

The present analysis demonstrated that the incidence of false positives in recurrent glioma surgery is not negligible. This is especially true when PP is considered for differential diagnosis. PP has been reported to occur predominantly (almost 60% of cases) within the first 3 months after completing adjuvant treatments, although it may occur later, as reported after medical administration of lomustine and temozolomide. In addition, methylguanine-DNA methyltransferase (MGMT) methylation tumor’s status has been associated with PP occurrence (21). Hence, if timing and molecular status are consistent, it is important for the neurosurgeon performing 5-ALA-guided resections to be aware of concerns regarding the accurate diagnosis of this phenomenon and that fluorescence status might hinder non-neoplastic tissue. On the other hand, in this setting, non-tumor-related 5-ALA positivity can be used as a guide to target surgical excision to areas of inflammatory infiltrations or reactive gliosis when surgery is indicated for edema relief.

Nevertheless, 5-ALA in the recurrent setting is valuable to the surgeon when RN is suspected: as demonstrated in the present analysis, 5-ALA-related fluorescence is rather more specific. Hence, this can provide surgeon guidance for proper histopathological sampling to increase diagnostic yield and tailor resection.

Indeed, 5-ALA can provide a feasible guidance especially in the recurrent setting, where MRI-based information often fails to preoperatively identify proper oncological tissue.

The “real-life” intraoperative picture corresponds to a substantial lack of textural feedback (tumor is often friable) and presence of scar, inflammation, neo-angiogenesis, and gliosis that further compromise a surgeon’s ability to distinguish tumor from non-tumoral tissue. This corresponds to a mixture of reactive/regressive tissue that shows areas with different degrees or absence of fluorescence along with proper neoplastic tissue that display fluorescence (17).

Therefore, a robust awareness of 5-ALA potentialities and pitfalls in recurrent glioma surgery is therefore paramount for a cognizant surgical strategy, especially when in proximity to eloquent brain areas where oncological benefit and functional cost associated with an aggressive resection should be considered.

## Conclusions

5-ALA is considered as one of the most valuable and widespread innovations in the field of HGG surgery. Its indisputable role has been well underlined for newly diagnosed HGG cases, whereas its significance, potentialities, and drawbacks in the recurrent setting are less clear.

The present study sheds light on 5-ALA’s possible role in recurrent glioma surgery, to appropriately guide surgical strategy, especially when PP or RN is suspected. Further clinical trials could confirm the results of the present meta-analysis.

## Data Availability Statement

The original contributions presented in the study are included in the article/**Supplementary Material**. Further inquiries can be directed to the corresponding author.

## Author Contributions

LR, CS, GDP, and AS contributed to conception and design of the study. VS, MA, and TS organized the database. TI and ST performed the statistical analysis. LR and CS wrote the first draft of the article. AR, MM, and GDP supervised the article. All authors contributed to article revision and approved the submitted version.

## Conflict of Interest

The authors declare that the research was conducted in the absence of any commercial or financial relationships that could be construed as a potential conflict of interest.

## Publisher’s Note

All claims expressed in this article are solely those of the authors and do not necessarily represent those of their affiliated organizations, or those of the publisher, the editors and the reviewers. Any product that may be evaluated in this article, or claim that may be made by its manufacturer, is not guaranteed or endorsed by the publisher.

## References

[1] Schupper AJ Yong RL Hadjipanayis CG . The Neurosurgeon's Armamentarium for Gliomas: An Update on Intraoperative Technologies to Improve Extent of Resection. J Clin Med (2021) 10(2):236. doi: 10.3390/jcm10020236 PMC7826675 33440712

[2] La Rocca G Della Pepa GM Menna G Altieri R Ius T Rapisarda A . State of the Art of Fluorescence Guided Techniques in Neurosurgery. J Neurosurg Sci (2019) 63(6):619–24. doi: 10.23736/S0390-5616.19.04854-9 31961115

[3] Schipmann S Muther M Stogbauer L Zimmer S Brokinkel B Holling M . Combination of ALA-Induced Fluorescence-Guided Resection and Intraoperative Open Photodynamic Therapy for Recurrent Glioblastoma: Case Series on a Promising Dual Strategy for Local Tumor Control. J Neurosurg (2020) 24:1–11. doi: 10.3171/2019.11.JNS192443 31978877

[4] Della Pepa GM Ius T La Rocca G Gaudino S Isola M Pignotti F . 5-Aminolevulinic Acid and Contrast-Enhanced Ultrasound: The Combination of the Two Techniques to Optimize the Extent of Resection in Glioblastoma Surgery. Neurosurgery (2020) 86(6):E529–40. doi: 10.1093/neuros/nyaa037 32186345

[5] Navarro-Bonnet J Suarez-Meade P Brown DA Chaichana KL Quinones-Hinojosa A . Following the Light in Glioma Surgery: A Comparison of Sodium Fluorescein and 5-Aminolevulinic Acid as Surgical Adjuncts in Glioma Resection. J Neurosurg Sci (2019) 63(6):633–47. doi: 10.23736/S0390-5616.19.04745-3 31961116

[6] Traylor JI Pernik MN Sternisha AC McBrayer SK Abdullah KG . Molecular and Metabolic Mechanisms Underlying Selective 5-Aminolevulinic Acid-Induced Fluorescence in Gliomas. Cancers (Basel) (2021) 13(3):580. doi: 10.3390/cancers13030580 33540759 PMC7867275

[7] Mazurek M Kulesza B Stoma F Osuchowski J Mandziuk S Rola R . Characteristics of Fluorescent Intraoperative Dyes Helpful in Gross Total Resection of High-Grade Gliomas-A Systematic Review. Diagnostics (Basel) (2020) 10(12):1100. doi: 10.3390/diagnostics10121100 PMC7766001 33339439

[8] La Rocca G Sabatino G Menna G Altieri R Ius T Marchese E . 5-Aminolevulinic Acid False Positives in Cerebral Neuro-Oncology: Not All That Is Fluorescent Is Tumor. A Case-Based Update and Literature Review. World Neurosurg (2020) 137:187–93. doi: 10.1016/j.wneu.2020.01.238 32058110

[9] Della Pepa GM Sabatino G la Rocca G . "Enhancing Vision" in High Grade Glioma Surgery: A Feasible Integrated 5-ALA + CEUS Protocol to Improve Radicality. World Neurosurg (2019) 129:401–3. doi: 10.1016/j.wneu.2019.06.127 31229752

[10] Lau D Hervey-Jumper SL Chang S Molinaro AM McDermott MW Philips JJ . A Prospective Phase II Clinical Trial of 5-Aminolevulinic Acid to Assess the Correlation of Intraoperative Fluorescence Intensity and Degree of Histologic Cellularity During Resection of High-Grade Gliomas. J Neurosurg (2016) 124(5):1300–9. doi: 10.3171/2015.5.JNS1577 26544781

[11] Utsuki S Oka H Sato S Shimizu S Suzuki S Tanizaki Y . Histological Examination of False Positive Tissue Resection Using 5-Aminolevulinic Acid-Induced Fluorescence Guidance. Neurol Med Chir (Tokyo) (2007) 47(5):210–3; discussion 213–4. doi: 10.2176/nmc.47.210 17527047

[12] Cozzens JW Lokaitis BC Moore BE Amin DV Espinosa JA MacGregor M . A Phase 1 Dose-Escalation Study of Oral 5-Aminolevulinic Acid in Adult Patients Undergoing Resection of a Newly Diagnosed or Recurrent High-Grade Glioma. Neurosurgery (2017) 81(1):46–55. doi: 10.1093/neuros/nyw182 28498936

[13] Panciani PP Fontanella M Schatlo B Garbossa D Agnoletti A Ducati A . Fluorescence and Image Guided Resection in High Grade Glioma. Clin Neurol Neurosurg (2012) 114(1):37–41. doi: 10.1016/j.clineuro.2011.09.001 21963142

[14] Kiesel B Mischkulnig M Woehrer A Martinez-Moreno M Millesi M Mallouhi A . Systematic Histopathological Analysis of Different 5-Aminolevulinic Acid-Induced Fluorescence Levels in Newly Diagnosed Glioblastomas. J Neurosurg (2018) 129(2):341–53. doi: 10.3171/2017.4.JNS162991 29076783

[15] Labuschagne JJ . 5-Aminolevulinic Acid-Guided Surgery for Recurrent Supratentorial Pediatric Neoplasms. World Neurosurg (2020) 141:e763–9. doi: 10.1016/j.wneu.2020.06.019 32526366

[16] Kamp MA Felsberg J Sadat H Kuzibaev J Steiger HJ Rapp M . 5-ALA-Induced Fluorescence Behavior of Reactive Tissue Changes Following Glioblastoma Treatment With Radiation and Chemotherapy. Acta Neurochir (Wien) (2015) 157(2):207–13; discussion 213-204. doi: 10.1007/s00701-014-2313-4 25547719

[17] Chohan MO Berger MS . 5-Aminolevulinic Acid Fluorescence Guided Surgery for Recurrent High-Grade Gliomas. J Neurooncol (2019) 141(3):517–22. doi: 10.1007/s11060-018-2956-8 30097823

[18] Sun YZ Yan LF Han Y Nan HY Xiao G Tian Q . Differentiation of Pseudoprogression From True Progressionin Glioblastoma Patients After Standard Treatment: A Machine Learning Strategy Combinedwith Radiomics Features From T1-Weighted Contrast-Enhanced Imaging. BMC Med Imaging (2021) 21(1):17. doi: 10.1186/s12880-020-00545-5 33535988 PMC7860032

[19] Zikou A Sioka C Alexiou GA Fotopoulos A Voulgaris S Argyropoulou MI . Radiation Necrosis, Pseudoprogression, Pseudoresponse, and Tumor Recurrence: Imaging Challenges for the Evaluation of Treated Gliomas. Contrast Media Mol Imaging (2018) 2018:6828396. doi: 10.1155/2018/6828396 30627060 PMC6305027

[20] Ellingson BM Chung C Pope WB Boxerman JL Kaufmann TJ . Pseudoprogression, Radionecrosis, Inflammation or True Tumor Progression? Challenges Associated With Glioblastoma Response Assessment in an Evolving Therapeutic Landscape. J Neurooncol (2017) 134(3):495–504. doi: 10.1007/s11060-017-2375-2 28382534 PMC7893814

[21] Li H Li J Cheng G Zhang J Li X . IDH Mutation and MGMT Promoter Methylation Are Associated With the Pseudoprogression and Improved Prognosis of Glioblastoma Multiforme Patients Who Have Undergone Concurrent and Adjuvant Temozolomide-Based Chemoradiotherapy. Clin Neurol Neurosurg (2016) 151:31–6. doi: 10.1016/j.clineuro.2016.10.004 27764705

