# Supplementary material for: 5-Aminolevulinic Acid False-Positive Rates in Newly Diagnosed and Recurrent Glioblastoma: Do Pseudoprogression and Radionecrosis Play a Role? A Meta-Analysis
Source: Front Oncol. 2022 Feb 17;12:848036. doi: 10.3389/fonc.2022.848036 (PMC8891510; doi:10.3389/fonc.2022.848036)
Supplement: Supplementary file 1 [file Table_1.docx]

| **Author and year** | **Reason(s) for exclusion** |
| --- | --- |
| Nabavi A. et al. 2009 | This study included grade III-IV glioma patients, and data from glioblastoma patients only were not retrievable |
| Kostron H. et al. 2010 | Patients’ data are not reported in the paper – Short communication |
| Dìez Valle R. et al. 2011 | This study included newly diagnosed and recurrent glioblastomas, but data from these two subgroups were not retrievable |
| Kallenberg et al. 2012 | Case report |
| Della Puppa A. et al. 2014 | This study included newly diagnosed and recurrent glioblastomas, but data from these two subgroups were not retrievable |
| Archavlis E. et al. 2014 | Data on fluorescence are not reported |
| Kamp M.A. et al. 2015 | This study included only patients operated for suspected recurrent glioblastoma but different histological findings after surgery |
| La Rocca G. et al. 2020 | No data on fluorescence or histological correlations |
| Barbagallo G.M.V. et al. 2021 | No data on fluorescence or histological correlations |
| Shipmann S. et al. 2021 | No data on fluorescence or histological correlations |
| Hingtgen S. et al. 2013 | Animal and in vitro study |
| Schucht P. et al. 2012 | This study included newly diagnosed and recurrent glioblastomas, but data from these two subgroups were not retrievable |
| Tykocki T. et al. 2012 | Included 5 patients |
| Hickmann A.K. Et al 2015 | Non comparative study |
| Quick-Weller J. Et al. 2016 | Non comparative study |
